# Evaluation of the Current Status of the Combinatorial Approach for the Study of Phase Diagrams

**DOI:** 10.6028/jres.117.018

**Published:** 2012-12-21

**Authors:** W. Wong-Ng

**Affiliations:** National Institute of Standards and Technology, Gaithersburg, MD 20899

**Keywords:** combinatorial approach, diffusion couple/diffusion multiple techniques, evaluation, phase diagrams, thin film phase diagram techniques

## Abstract

This paper provides an evaluation of the effectiveness of using the high throughput combinatorial approach for preparing phase diagrams of thin film and bulk materials. Our evaluation is based primarily on examples of combinatorial phase diagrams that have been reported in the literature as well as based on our own laboratory experiments. Various factors that affect the construction of these phase diagrams are examined. Instrumentation and analytical approaches needed to improve data acquisition and data analysis are summarized.

## 1. Introduction

### 1.1 Use and Preparation of Conventional Phase Diagrams

Phase diagrams are critical research tools for many scientific disciplines including materials science, ceramics, geology, physics, metallurgy, chemical engineering and chemistry. Phase diagrams contain important information for the development of new materials, control of structure and composition of critical phases, and improvement of properties of technologically important materials. These diagrams often can be thought as ‘road maps’ for processing as they provide the theoretical basis for synthesis of materials. Applications of phase diagrams range from preparation of high-quality single crystals and single-phase materials to controlled precipitation of second phases and formation of melts.

Before any material synthesis or processing, it is logical to first study the relevant phase diagrams to obtain information about the phase relationships and other characteristics of the material. Phase diagrams can be used to obtain a wide range of information, such as (1) identity of compounds formed from specific end members, (2) temperature/composition stability range of a specific phase, (3) optimum conditions for synthesis, (4) impurities likely to be present, (5) region of homogeneity of solid solutions, (6) polymorphism of a phase, (7) crystallization field of a phase, (8) understanding of melt evolution, (9) effect of temperature and pressure on processing, and (10) quantitative information for the preparation of controlled mixtures. Additionally, phase diagrams are invaluable for designing a systematic approach to ‘discover’ new phases with desirable properties.

As phase diagrams provide critical information for the scientific community, there are various world centers that are responsible for the compilation of these diagrams. For over 60 years, the American Ceramic Society (ACerS), in collaboration with NIST, has evaluated and published the Phase Diagrams for Ceramists series of compilations. Since the cooperative program between NIST and ACerS was formalized in 1982, computerization of data was established as a major goal to modernize the book products [1]. More than 24,000 entries have been published in 23 book volumes and in the CDROM products. The continuous growth rate is about 800 new commentaries (with 1200 phase diagrams) per year. These products have provided high-quality phase equilibrium data to the technical community. A parallel program in alloy phase diagrams is currently managed by the American Society for Metals (ASM) [2]. The ASM Alloy Phase Diagrams Center is copyrighted by ASM International, and the data in this product are copyrighted by Material Phases Data System, Vitznau, Switzerland. A portion of the binary diagrams is jointly copyrighted by Material Phases Data System and Japan Science and Technology Corporation, Tokyo, Japan.

The preparation of an equilibrium phase diagram is a long process that requires a committed effort using a combination of methods [3,4]. For example, to construct subsolidus phase diagrams, a number of compositions are prepared in the region of interest, followed by equilibration and determination of the phase assemblages present. Homogeneity regions and tie-lines connecting phases in equilibrium are determined by studying the evolution of these compositions as a function of time, temperature, and pressure (including partial pressures of volatile species). The experimental methods for determining phase diagrams are generally divided into two types: static and dynamic. In the static methods, the temperature of the sample is held constant until equilibrium is attained. The most widely used of these is the quenching method (air quench or quench into a medium such as water bath, oil, or liquid-nitrogen-cooled helium). Quenching studies in combination with powder X-ray diffraction and petrographic microscopy for phase identification are essential for obtaining subsolidus phase diagrams. Liquidus phase diagrams require further experiments for determining the melting temperatures of phases or assemblages and the estimation of liquid compositions. Dynamic techniques, including differential thermal analysis (DTA) and thermogravimetric analysis (TGA), are needed to obtain an indication of phase transitions, reactions, and associated loss of volatiles, such as oxygen. Electron-beam based methods such as transmission electron microscopy (TEM) and scanning electron microscopy (SEM) are used to further define the microstructures of the experimental products, particularly of the crystalline phases. Quantitative micro-beam analytical techniques, including energy dispersive x-ray analysis (EDS) or wavelength dispersive methods (WDS), are used for the determination of the compositions of both the crystalline phases and the quenched liquid.

For both conventional phase diagrams and the combinatorially constructed phase diagrams discussed below, it should be noted that in principle it is desirable to demonstrate reversibility of each equilibrium represented. In practice this is not always possible because of kinetic limitations, with the exception of solid/liquid equilibria. It is often assumed that equilibrium is reached if no further changes occur with increased annealing times. While this may be a reasonable assumption, it does not constitute proof of the most stable equilibrium. In general, attainment of the thermodynamically most stable state is difficult to prove, as there may be kinetically hindered phases which are not readily observed experimentally, yet would provide the thermodynamically most stable assemblage. Nonetheless, a carefully determined phase diagram is still a useful representation of equilibrium, even though portions may represent metastable equilibrium. In situations where phases have been reported in the literature, but are not present on the phase diagram, the possibility of metastable equilibria should be considered.

### 1.2 Combinatorially Constructed Phase Diagrams

Clearly, constructing a traditional phase diagram using a one-sample-at-a-time approach with the traditional equilibrated alloy or ceramic method is a labor intensive endeavor that could require months or years of effort. Because of the extensive use of thin film technology, although information from conventional equilibrium diagrams is indisputably important as a base-line for comparison, there is a need for diagrams relevant to as-processed thin films. High-throughput combinatorial approaches to the study of alloys include synthesizing a sample with an array of alloy or ceramic compositions (material libraries) and then studying/screening their structures and properties simultaneously or serially. In principal, this offers a substantial reduction in time relative to the commonly used one-sample-at-a-time approach.

Although the combinatorial high-throughput approach appears to be convenient for studies of phase diagrams, it is a relatively new approach, with associated pitfalls that need to be considered. The following serves as a summary of various factors that would affect the construction of some of these phase diagrams, as evidenced by the literature data as well as experience from this laboratory. This report is not intended to serve as an exhaustive review of the theory and techniques for studying phase diagrams or of the combinatorial approach to materials science, but rather as an organized collection of observations that may prove useful to those initiating research in these fields.

According to Zhao [5], attempts to accelerate phase diagram studies combinatorially can be traced back at least half a century ago. In 1955, Boettcher et al. [6] designed and deposited thin films with composition gradients, and used electron diffraction to identify phases, in order to construct a phase diagram of the Ag-Pb-Sn system. A decade later, Kennedy et al. [7,8] used a similar approach to map the Cr-Fe-Ni ternary phase diagram. Recently developed state-of-the-art methods for phase diagram mapping using thin film deposition include “continuous phase diagramming [5,9]” and the “phase diagram on a chip [10,11].” Improvements associated with these methods were found to be mainly due to excellent computer control, compositional masking schemes for the thin films, and improved probes for measurement of micro-scale crystal structure and properties. In addition to the thin-film approach, diffusion couples and diffusion multiples, which can be regarded as a form of combinatorial investigation, have been used in the field of metallurgy for many decades for constructing phase diagrams of intermetallic systems [5,12–19].

## 2. Combinatorial Methods for Construction of Phase Diagrams

In constructing any phase diagram, whether oxide or alloy, it is necessary to answer the following questions: (1) What are the most effective ways of preparing the diagram/library, (2) What are the best techniques to analyze the compositions so as to ascertain the phases present and construct the tie-line relationships, (3) How can it be determined whether the result represents equilibrium at the given conditions; how well does it match the equilibrium diagram prepared using bulk methods; how does it compare with the calculated diagram; (4) How well does the library reproduce the relevant properties of the bulk materials; (5) How can the property information collected for the library be rapidly assessed.

In the following we will discuss two approaches for constructing combinatorial phase diagrams. The first utilizes the diffusion couple/diffusion multiple technique; the second approach uses thin films prepared by various deposition techniques.

### 2.1 Diffusion Couple/Diffusion Multiple Technique for Intermetallic Systems

#### 2.1.1 Background

The idea of using a diffusion couple materialized in the early 1960s as a result of a number of reports on multiphase diffusion, solid state bonding, and applications of electron probe microanalysis (EPMA) to the study of interfacial reactions. In recent years, there has been significant progress in the technique, due to the advances of the expanded/evolved technique referred to as “diffusion multiples.” The diffusion-multiple approach [13–16] was an outgrowth of both the diffusion couple method and the ‘ternary diffusion couple’ method introduced by Hasebe and Nishizawa [18] and by Jin et al. [19]. Zhao [15,20] is credited for his leadership in this development by applying a process using hot isostatic pressing (HIP) method which allows multiple diffusion couples and triples to be included in a single sample, thus creating a diffusion multiple. During the development of the diffusion multiple method, it was realized that localized property measurements allowed property mapping in addition to the construction of phase diagrams. Furthermore, diffusion couples and triples made up of multicomponent alloys were very useful for discovering new alloys.

According to Zhao et al. [13–16, 20], the use of the diffusion couple in phase diagram studies is based on the assumption of local equilibrium at the phase interfaces in the diffusion zone. This implies that an infinitesimally thin layer adjacent to the interface in such a diffusion zone is in thermodynamic equilibrium with its neighboring layer on the other side of the interface. A diffusion multiple is an assembly of three or more different metal blocks, in intimate interfacial contact, and subjected to a high temperature to allow thermal interdiffusion. Ideally, the interdiffusion among the elements forms all the intermetallic compounds and solid-solution phases in that multi-component system, creating complete libraries of the single-phase compositions. In other words, the existence and establishment of the local equilibrium at phase interfaces in the diffusion zone is the basis for applying diffusion multiples to phase diagram determination. He further assumed that local equilibrium is maintained in the diffusion zone, which means that reaction is very rapid compared to the rate of diffusion.

#### 2.1.2 Sample Analysis Techniques for the Diffusion Couple Approach [14]

The availability of advanced micro-analytical tools such as electron back scatter diffraction (EBSD), and electron probe microanalysis (EPMA) has made the use of diffusion multiples a powerful tool for phase diagram studies.

1. Electron probe microanalysis (EPMA)
  EPMA is a technique used to measure concentration profiles in diffusion couples since the 1960s. In EPMA, high energy electrons are focused to a fine probe and directed at the spot of interest in the diffusion couple/multiple. The incident electrons interact with the atoms in the sample and generate, among other signals, characteristic x-rays. These x-rays are detected and identified, and with the use of suitable standards they can be corrected for matrix effects in order to perform quantitative analysis. The chemistry of volumes of 1 μm dia. or less, depending on the elemental composition and sample geometry, can be sampled
2. Electron back scattering diffraction (EBSD)
  EBSD is applicable to most combinatorial inorganic thin film and diffusion multiple samples. Commercial EBSD systems often are used as an attachment to regular scanning electron microscopes. As a focused electron beam impinges on a sample, it generates backscattered electrons in addition to secondary electrons, Auger electrons, x-rays and others. The backscattered electrons escaping the samples have been scattered/diffracted by the crystal lattice, thus producing a spatially and angularly resolved intensity variation. When an area detector is used to capture the backscattered electrons, a pattern similar to a Kikuchi map is obtained. Phase identification is accomplished by a direct match of the diffraction bands in an experimental EBSD pattern with simulated patterns generated using known structure types and lattice parameters. However, EBSD has two drawbacks. First, it cannot provide accurate cell parameter measurements. Second, with EBSD, it is sometimes difficult to differentiate certain crystal structures with closely related space group relationships.

#### 2.1.3 Literature Diagrams Constructed by the Diffusion Couple/Multiple Technique

Zhao and his collaborators have summarized methods for the design and fabrication of metal alloy diffusion multiples, and have discussed effective analysis using EPMA and electron back scattering diffraction, and extraction of phase diagram data from EPMA results, as well as strategies for dealing with some of the limitations of the diffusion multiples approach.

**Fig. 1:**
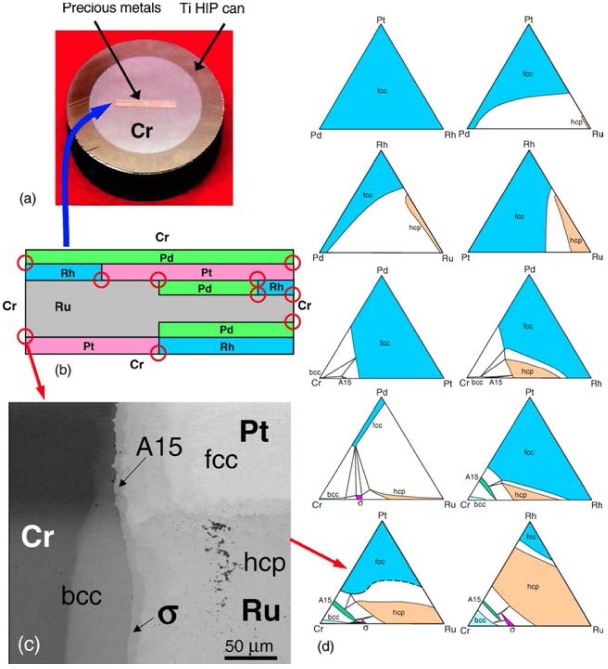
A diffusion multiple for rapid mapping of ternary phase diagrams in the Pd-Pt-Rh-Ru-Cr system. (a) optical image of the sample; (b) arrangement of precious metal foils in the diffusion multiple to create many tri-junctions shown in circles; (c) BSE image of the Cr-Pt-Ru tri-junction showing the formation of the A15 (Cr_3_(Pt,Ru)) and σ (Cr_x_(Ru,Pt)_y_) phases due to interdiffusion of Pt, Ru, and Cr as well as electron microprobe scan location (lines); (d) ten ternary phase diagrams (isothermal sections at 1200 °C) obtained from this single diffusion multiple. The phase diagrams are plotted on atomic percent axes with the scales removed for simplicity [13,17]. (Reprinted with permission from [17]. © 2004 Springer)

**Fig. 2:**
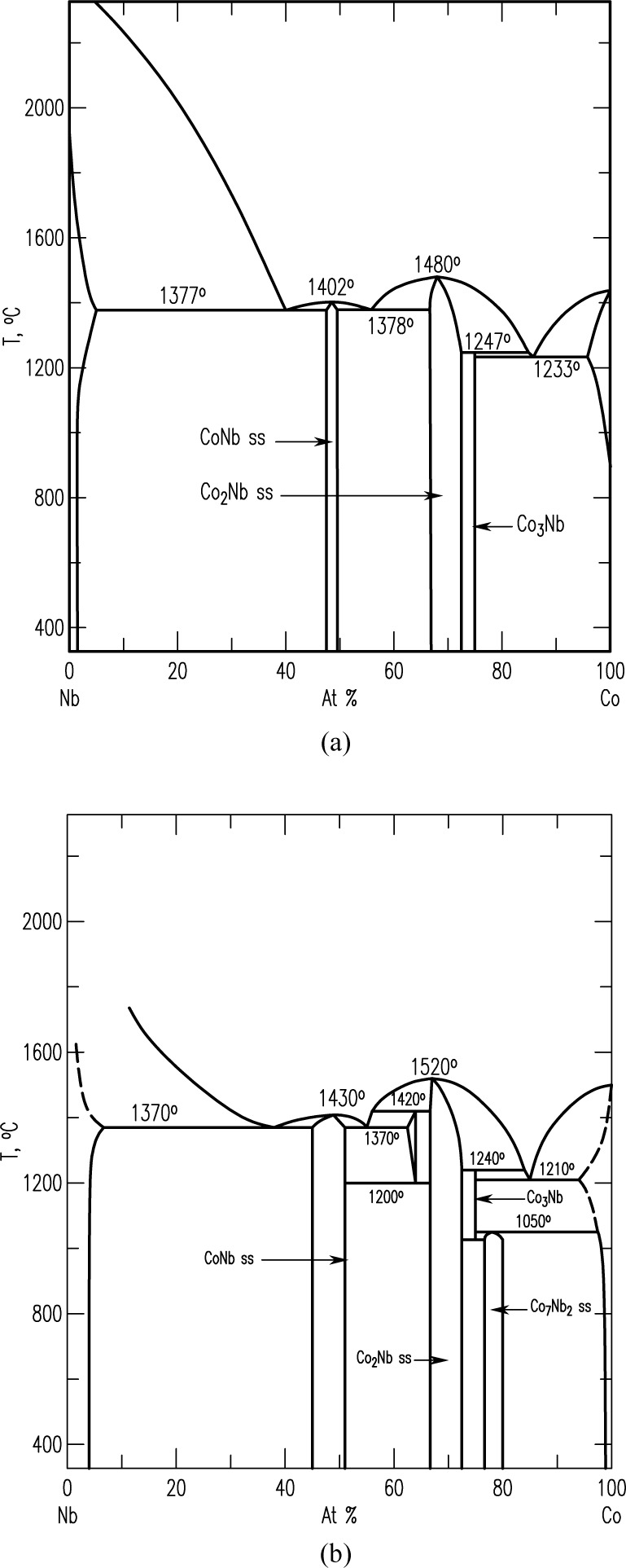
Phase diagrams of the Co-Nb system obtained by different equilibration techniques, (a) by Massalski et al. [23] and (b) by Brande et al. [24].

**Fig. 3:**
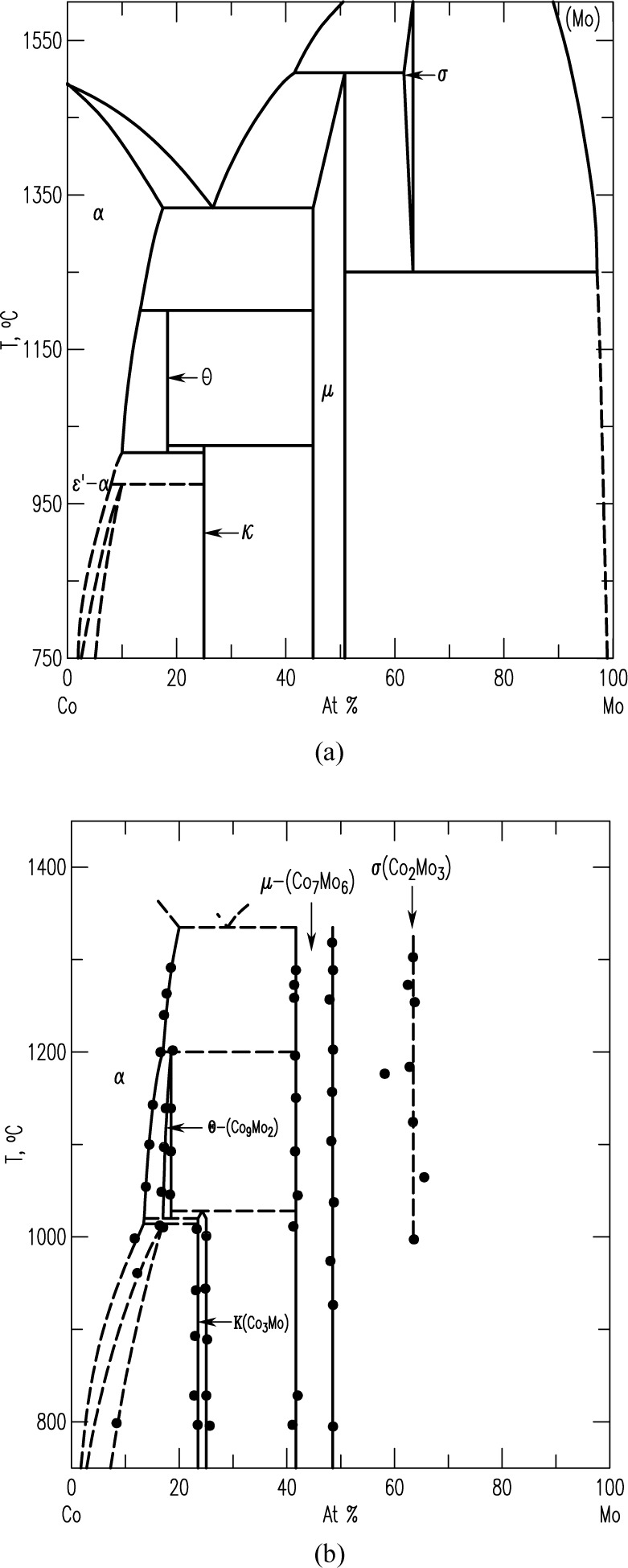
(a) Equilibrium phase diagram of the Mo-Co system; (b) Diagram obtained using diffusion couple approach [25].

**Fig. 4:**
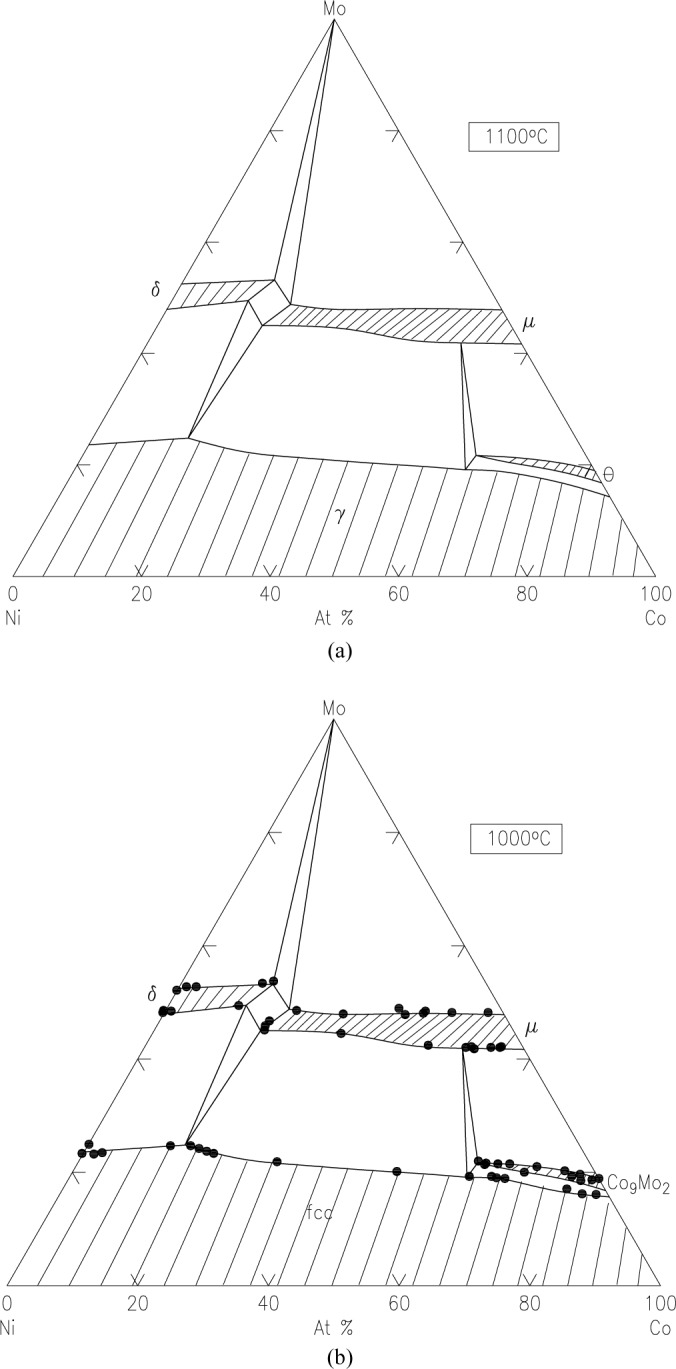
(a) Isothermal section of the phase diagram of the Co-Mo-Ni system as 1100 °C obtained by the diffusion multiple approach, (b) by the equilibration approach [5].

**Fig. 5:**
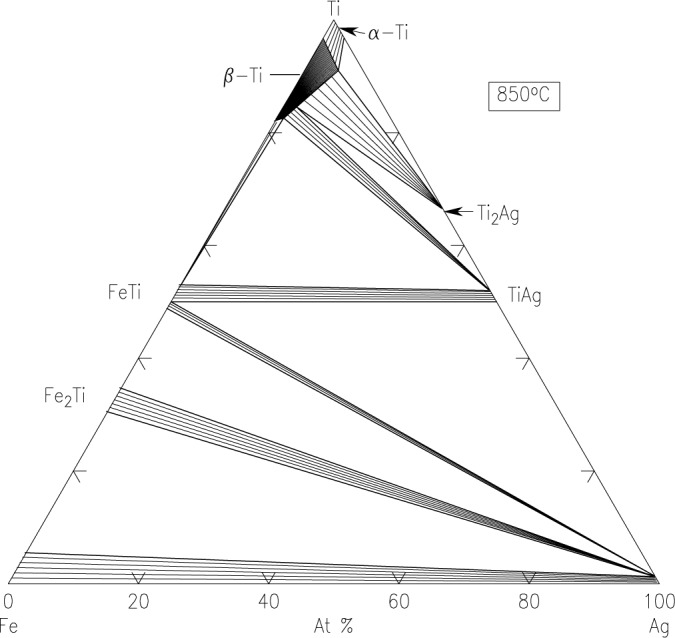
The Ti-Ag-Fe system at 850 °C obtained by the diffusion multiple approach and by equilibrated samples [29,30].

The applicability of the diffusion-multiple approach to the determination of complex intermetallic systems has been demonstrated by research on more than 30 systems [5,13–17, 22–41]. Examples include Mo-Co-Cr [5,14], Pd-Pt-Rh-Ru-Cr [13], Co-Mo-Ni [17], Co-Nb [22], Fe-Mo-Ni [21,26], Ti-Ag-Fe [29,30], Nb-Cr-Si [17,37,38], Ni-Nb [22], Mo-Co [25], Al-Cr-Ti [27], Ag-Sn-Zn [28], Co-Cu-Ti [31], Nb-Ti-Si [32–34], Ti-Ni-Cr [39,40], Nb-Ni-½N_2_, Mo-Si-½N_2_ (*f*_N2_ < 1.8×10^−4^ (fugacity)), Mo-Si-½N_2_ (1.8×10^−4^ < *f*_N2_ < 0.03), Mo-Si-½N_2_ (0.03 < *f*_N2_ < 7), Mo-Si-½N_2_(7 < *f*_N2_ < 88), Mo-Si-½N_2_ (88 < *f*_N2_ < 98, Mo-Si-½N_2_ (*f*_N2_ > 98) [39]. In many cases, the diagrams constructed using the diffusion couple/multiple approach are similar to those obtained using the equilibrium approach. In other cases, minor differences were found. Among these systems, we selected six examples for illustration:

1. Pd-Pt-Rh-Ru-Cr system (including subsystems Pt-Pd-Rh, Pt-Pd-Ru, Rh-Pd-Rh, Rh-Pt-Ru, Pd-Cr-Pt, Pd-Cr-Rh, Pd-Cr-Ru, Pt-Cr-Rh, Pt-Cr-Ru, Rh-Cr-Ru)
  The best demonstration of using the diffusion multiple approach for creating phase diagrams is the Pd-Pt-Rh-Ru-Cr system (Figs. 1a–1d) as investigated by Zhao et al. [13,17]. The diffusion multiple was prepared by cutting a slot 1.8 mm side and 12.7 mm long from a 25 mm diameter pure chromium disk of 3 mm thickness. Pure Pd, Pt, and Rh foils of 0.25 mm thickness were arranged in the geometry as shown in Figs. 1a and 1b and then put into the slot in the Cr disk along with a pure Rh piece with two steps in it. The Rh piece had a thickness of 1 mm on one side and 0.5 mm on the other. The entire assembly was placed in a hot isostatic press (HIP) for heat treatment at 1200 °C, 200 MPa for 4 h, then further annealed at 1200 °C for 36 h. The interdiffusion of elements at the tri-junction regions of the diffusion multiple allows the formation of all the intermediate compounds and the generation of composition variations for all the single phase regions. An example of a specific location, where Cr, Pt, and Ru met in the tri-junction, is shown in Fig. 1c. Interdiffusion of Cr and Pt formed the A15 phase (Cr_3_(Pt, Ru), and that of Cr and Ru formed the σ(Cr_y_(Ru,Pt)_x_) phase; close to the tri-junction region, ternary diffusion took place. Ten phase diagrams (isothermal sections at 1200 °C for 40 h) were obtained using compositional information from EPMA and the structure information from EBSD (Fig. 1d). In summary, it was possible to map isothermal phase diagrams of ten ternary systems using only a single diffusion multiple; using conventional methods, it would probably take 1000 alloys to map these corresponding diagrams. The efficiency gain is tremendous.
2. Co-Nb
  Two diagrams of the Co-Nb system have been reported, one by Massalski [23] and the other one by Brandes [24] (Figs. 2a and 2b, respectively). There are noticeable discrepancies between these two diagrams; for example, different numbers of phases are present (the Co_7_Nb_2_ phase does not exist in Fig. 2a), and the solid solution ranges differ [22]. Later studies of the system using a multiphase diffusion approach, with optical metallography and electron microprobe analysis as characterization tools, allowed identification of the phases present in the diffusion zones of the Co-Nb diffusion couples, in agreement with the diagram reported by Brandes (Fig. 2b) [24].
3. Mo-Co
  The equilibrium diagram of the system Mo-Co was reported by Quinn and Hume-Rothery [14] (Fig. 3a). More recently, the phase diagram was investigated between 800 °C and 1300 °C (Fig. 3b) by microscopy and X-ray diffraction using diffusion couples consisting of pure metals and their alloys (by Heuwegen et al. [25]). There are small discrepancies between the two diagrams primarily in the extent of the solid solution of the σ phase. Heuwegen et al. concluded that the σ layer is too thin for an accurate determination of the phase boundary concentrations. This illustrates the possibility of missing phases in the diffusion couple (or multiple) approach, especially at low temperatures where kinetic factors may come into play.
4. Co-Mo-Ni
  Zhao et al. [17] compared the 1100 °C isothermal section of the Co-Mo-Ni ternary system which was constructed using the equilibrated alloys approach (Fig. 4a) with that prepared using the diffusion approach (Fig. 4b). An excellent agreement of the diagrams prepared by the two different approaches is apparent.
5. Ti-Ag-Fe
  The Ti-Ag-Fe system was studied at 850 °C by Kodentsov [29] and van Beek et al. [30]. This system is of considerable practical importance because Ag-Ti alloys are widely used as active filler metals for brazing components. All equilibria found in the multiphase diffusion couples are consistent with the ternary Ag-Fe-Ti system given in Fig. 5. The experimentally established phase relations were confirmed by examining a number of ternary equibrated alloys and, in addition, they were verified by a thermodynamic evaluation of this system.

A number of other diagrams constructed using the diffusion-multiples method have been compared with diagrams from equilibrated alloys (for example, Fe-Ni-Mo, Ni-Co-Mo, Pt-Cr-Ru and Cr-Al-Ti) [17,26,27,41]. In general, good agreement was observed between the phase equilibria in the isothermal sections obtained by the two methods. The conclusion after these comparisons is that the phase diagrams determined from diffusion couples/multiples are reliable. These phase diagram techniques, in principle, could also be applied to ceramics such as the oxide systems. The following summarizes the advantages and limitations of the Diffusion Couple/Multiple technique.

#### 2.1.4 Advantages and Limitations of the Diffusion Couple/Multiple Technique [14,15,17]

##### 2.1.4.1 Advantages

1. The efficiency gain using the diffusion couple/multiple technique as compared to conventional one-alloy-at-a-time (equilibrated alloy) phase diagram methods determination is immense, even though a substantial amount of effort is involved in preparing the couples. Since an entire phase diagram can be obtained from a tri-junction region of a diffusion multiple, by creating several tri-junctions on one sample, isothermal sections of multiple ternary systems can be determined without having to make dozens or even hundreds of alloys. Using conventional methods, it took Goldschmidt and Brand about 220 alloys to determine the Nb-Cr-Si ternary system [42] and similarly about 135 alloys were used by Lysenko et al. [43] to determine the Ti-Cr-Si system.
2. The economic advantage of using the diffusion couple/multiple technique is also substantial in terms of the raw materials usage; this becomes especially important where high-purity samples of precious or rare metals are required.
3. Using the traditional methods, one often encounters the problem of intergranular contamination. Since using the diffusion multiple approach, one only needs a few cast alloys, the avoidance of intergranular contamination is relatively easy (for example, by using Ta foil and quartz tubes filled with argon to contain the samples).
4. Formation of multiple phases in a single sample would save the subsequent analysis time, particularly those that require vacuum systems for analysis (for example, EPMA)-thus saving considerable effort in sample mounting as well.
5. In the diffusion multiples, melting or powder contamination would not be problems since all the phases are formed by diffusion reaction of bulk constituents at the temperature of interest. In principal, the phases formed should all be equilibrium phases and local equilibrium at the phase interface would give the equilibrium phase diagram.

##### 2.1.4.2 Limitations of the Diffusion Multiple Techniques

1. Long term annealing is required to grow the phases to sufficient thickness for quantitative analysis and structure determination. In some cases, the diffusion rate could be slow enough to prevent the formation of the required thicknesses.
2. Occasionally there may be missing phases in experiments conducted at low temperatures; there may be sluggish kinetics (especially nucleation difficulties) which require a long time. One may need to anneal the samples at high enough temperature for promoting inter-diffusion.
3. It is difficult to generate liquid-solid phase equilibrium information using the diffusion/multiple technique. It is best applied to subsolidus situations,and melting should be avoided.
4. There are often difficulties associated with accurate determination of phase boundary concentrations in the reaction zone. Determination of a chemical composition wit EPMA has an inherent experimental error associated with data counting statistics and data correction procedures. Accurate microprobe analysis near the interfaces is sometimes difficult owing to fluorescence and beam-spreading effects. In addition, there may be formation of a quasi-equilibrated diffusion zone, or formation of impurity stabilized phases.
5. Formation of a non-equilibrium phase in a diffusion zone is sometimes possible (for example, a phase that is stabilized by impurities present in the end-members). Therefore one should keep the impurity level as low as possible because an impurity could induce stabilization of metastable phases.
6. These diffusion methods would benefit from improvement of spatial resolution of the microanalytical tools. Errors attributable to extrapolated interface compositions can be minimized if steeper concentration gradients can be measured.
7. In some situations, one or more of the phases may not form by interdiffusion reactions, particularly at low temperatures. Therefore it is recommended that multiples be annealed at greater than half the melting temperatures (the solidus of the binary and ternary systems). One should compare data with selected equilibrated alloys. Combining results with theoretical modeling [44–46] is also beneficial.
8. The major source of errors lies in the interpretation and extraction of equilibrium tie-lines from EPMA data. Much information is condensed in a very small area of a sample. Although one cannot perform EPMA analysis at every location in the interdiffusion zone, one must avoid large scan step sizes.

### 2.2 Thin Film Techniques

#### 2.2.1 Background

In this paper, “thin films” generally refers to films of less than one micrometer thickness, with thickness greater than a few atomic monolayers, such that surface effects are not dominant, and the parameters that determine the phase equilibria are similar to those for the bulk. It is to be expected, however, that in some situations, there will be differences relative to the bulk because of the energetic nature of deposition processes and the effect of substrate epitaxy.

A number of the early combinatorial attempts involved thin films. In 1955, Boettcher et al. [6] deposited thin films with composition gradients and used electron diffraction to identify existing phases for constructing phase diagrams. They tested the methodology on the Ag-Sn and Au-Sn binary systems, and then applied the methodology to map the phase diagram of the Ag-Pb-Sn ternary isothermal section. The diagrams compared well with those calculated from thermodynamic modeling. A decade later, Kennedy et al. [8] used a similar approach to map the Cr-Fe-Ni ternary phase diagram by evaporating pure Cr, Fe, and Ni from three different ingots using electron-beam physical vapor deposition (EB-PVD). A thin film with varying ternary compositions on a substrate was created. The substrate was heated to 760 °C. However, despite overall matching of topology, some details, such as the locations of phase boundaries were different, as compared to the equilibrium diagram. This discrepancy was considered to be due to errors in composition determination by x-ray fluorescence (XRF).

The state-of-the-art combinatorial methods [9–11] follow the same general concepts introduced by Boettcher et al. [6] and Kennedy et al. [8]. Currently, three techniques are commonly used to create thin film combinatorial libraries. The first technique is known as sequential deposition with masking, either discrete or continuous [10]. New masking schemes have been developed to create complete ternary composition libraries [47,48]. The discrete combinatorial synthesis (DCS) approach was pioneered by Schultz and Xiang [49]. In this technique, sets of discrete chemical entities are generated using rapid sequential synthetic techniques. This approach is most suited for the investigation of biologically active molecules. DCS can be performed without the use of masks and thus at growth conditions comparable to those that yield optimized materials properties. The second method is the continuous composition spread (CCS) co-deposition method [50,51]. The CCS approach is based on a synthetic technique in which multiple elements are co-deposited on a substrate simultaneously from two or more sources that are chemically distinct. The film thus produced has an inherent composition gradient [8]. CCS syntheses are in general achieved using physical vapor deposition techniques such as evaporation and sputtering. An advantage of the CCS technique is that intimate mixing is guaranteed and that very fine compositional resolution can be achieved. The third method is called composition-gradient molecular-atomic layer epitaxy [52] which produces atomically controlled layer-by-layer growth of thin films with exact compositional and structure control. A scanning reflection high-energy electron diffraction (RHEED) system is typically used to perform *in situ* monitoring of the layer-by-layer epitaxy.

### 2.2.2 Techniques for Depositing Combinatorial Films [53]

1. Sputtering Deposition
  Sputtering deposition is a physical vapor deposition (PVD) method of depositing thin films by first ejecting material from a target, then deposits onto a substrate. The sputtered ions can ballistically travel from the target to the substrate in a straight line. Alternatively, at higher gas pressures, the ions collide with the gas atoms that act as a moderator and move diffusively and condense on the substrate. The sputtering gas is often an inert gas such as argon. For efficient momentum transfer, the atomic weight of the sputtering gas should be close to the atomic weight of the target. For sputtering light elements neon is preferable, while for heavy elements krypton and xenon are often used.
2. Pulsed Laser Deposition (PLD)
  PLD is a PVD thin film deposition technique where a high power pulsed laser beam is focused inside a vacuum chamber. As it strikes a target, the target material is vaporized as a plasma plume which then deposits as a thin film on a substrate. This process can occur in ultra high vacuum or in the presence of a background gas, such as oxygen. PLD has several key advantages over other techniques in library fabrication. Laser ablation of materials of bulk targets is a highly non-equilibrium process which allows stoichiometric transfer and delivery of target composition to the substrate. By monitoring the number of laser pulses, one can control the deposition of materials at an atomic layer level. Furthermore, by incorporating a layer-by-layer deposition technique, one can design and explore novel materials systems that may not exist in bulk forms.
3. Molecular Beam Epitaxy (MBE)
  In solid-source MBE, ultra-pure elements are heated in separate quasi-Knudsen effusion cells until they begin to slowly sublimate. The process takes place in high vacuum or ultra high vacuum (10^−8^ Pa). The evaporated atoms do not interact with each other or vacuum chamber gases until they reach the wafer due to the long mean free paths of the atoms. During operation, RHEED is often used for monitoring the growth of the crystal layers. A computer is used to control shutters in front of each furnace, allowing precise control of the thickness of each layer down to a single layer of atoms. One can fabricate a library of artificially designed crystal structures, superlattices, or nano-structured devices using this technique.
  In a small number of research laboratories, combined instrumentation also exists. For example at the National Institute of Materials Science (NIMS) in Japan and at the Ceramics Division of NIST, the sputtering system and the PLD unit are connected together via a vacuum chamber. Transferring of samples from one unit to the other can readily be accomplished through the special chamber without breaking the vacuum. Application of this facility includes deposition of metallic protection layers (using sputtering) on the surface of an oxide PLD thin film, and the study of material systems with mixed components of oxides and metals.

### 2.2.3 Compositional Analytical Tools for Combinatorial Thin Films [5,53]

Obtaining accurate mappings of composition and phase distribution is essential for constructing reliable relationships among composition, structure and properties of materials. However, it is easy to overlook the importance of the diagnostics in the combinatorial experiments. The original composition variation may shift or change during the processing, depending on the synthesis procedure or nature of the sample.

Presently techniques such as X-ray photoelectron spectroscopy (XPS), energy dispersive spectrometry (EDS), and wavelength dispersive spectrometry (WDS) can be used to collect X-ray spectra from samples being analyzed. These systems have specific advantages and disadvantages and the choice will depend on the materials studied. Electron or ion microprobes and X-ray fluorescence (XRF) have also been used for thin film composition mapping. The following summarizes some of these techniques.

1. X-Ray Photoelectron Spectroscopy (XPS)
  X-ray photoelectron spectroscopy (XPS) is a surface chemical analysis technique that can be used to analyze the surface chemistry of a material. It is a quantitative spectroscopic technique that measures the elemental composition, empirical formula, chemical state and electronic state of the elements that exist within a material. XPS spectra are obtained by irradiating a material with a beam of x-rays while simultaneously measuring the kinetic energy and number of electrons that escape from the top 1 to 10 nm of the material being analyzed. XPS requires ultra high vacuum conditions. The XPS technique detects all elements with an atomic number (Z) of 3 (lithium) and above. Detection limits for most of the elements are in the parts per thousands range.
2. Scanning Electron Microscopy (SEM/EDS)
  In scanning electron microscopy (SEM), an electron beam is scanned across a sample’s surface. When the electrons strike the sample, a variety of signals are generated, and it is the detection of specific signals which produces an image or a sample’s elemental composition. The three signals which provide the greatest amount of information in SEM are the secondary electrons, backscattered electrons, and X-rays. Energy Dispersive X-ray Spectroscopy (EDS) can provide rapid qualitative, or with adequate standards, quantitative analysis of elemental composition with a sampling depth of up to 1 to 2 µm. The EDS characteristics that are important with respect to quantitative analysis of combinatorial films are: (1) elemental detection for atomic number Z ≥ 11 (in routine applications), or Z ≥ 6 with windowless ultra-thin window detectors; (2) maximum x-ray energy resolution approximately 140 eV at the Mn_α_ line; (3) a low count rate and a low peak to background ratio, thus relatively low accuracy; (4) detectability limit on the order of 1000 ppm, and (5) difficulty in obtaining very accurate compositions. The advantages include simultaneous detection of all elements for faster analysis and relatively inexpensive equipment cost.
3. Wavelength Dispersive X-Ray Spectroscopy (WDS or WDXRF)
  The Wavelength dispersive X-ray spectroscopy (WDS or WDXRF) is a method used to count the number of X-rays of a specific wavelength diffracted by a crystal (obeying the Bragg’s law). WDS reads or counts only the x-rays of a single wavelength. Therefore the element must be known (or suspected to be present) in order to select the proper crystal capable of diffracting its characteristic radiation. The WDS characteristics that are important with respect to quantitative analysis of combinatorial films are: (1) elemental detection for atomic number Z ≥ 4; (2) X-ray energy resolution as good as 5 eV; (3) a high counting rate and low background which gives a detectability limit on the order of 100 ppm; and (4) higher accuracy in the composition than EDS. The disadvantages of WDS as compare to EDS include: (1) sequential detection of the elements requires longer analysis time; (2) much higher equipment cost, and (3) much higher beam current required.
4. Electron or Ion Microprobe
  The microprobe is an instrument that applies a stable and well-focused beam of electrons or charged particles to a solid sample to determine its spatially resolved elemental composition. The chemical composition of the target can be found from the elemental data extracted through emitted X-rays or measurement of an emitted secondary beam of material sputtered from the target. When the primary beam consists of accelerated electrons, the probe is called an Electron Microprobe, and when the primary beam consists of accelerated ions, the term Ion Microprobe is used. An Ion Microprobe produces a plasma from a small portion of the material; the analysis is done by the same basic techniques as the ones used in mass spectrometry.
5. X-Ray Fluorescence (XRF)
  The XRF method is fast and non-destructive to the sample and is widely used for elemental analysis and chemical analysis. When materials are exposed to short-wavelength X-rays or to gamma rays, ionization of their component atoms may take place. Following removal of an inner electron by an energetic photon provided by a primary radiation source, an electron from an outer shell drops into its place. Each of these transitions yields a fluorescent photon with a characteristic energy equal to the difference in energy of the initial and final orbital. The fluorescent radiation can be analyzed either by sorting the energies of the photons (energy-dispersive analysis) or by separating the wavelengths of the radiation (wavelength-dispersive analysis). Once sorted, the intensity of each characteristic radiation is directly related to the amount of each element in the material. By comparison with the electon microbeam-based EDS and WDS methods, the signal is generally collected from a much larger volume of material.

#### 2.2.4 Phase Identification and Structure Analysis Analytical Tools

For obtaining crystallographic data information combinatorial materials libraries, both conventional and synchrotron X-ray diffraction have been used. For example, a scanning laboratory X-ray microdiffractometer called the general area detector diffraction system (GADDS), manufactured by Bruker-AXS, has been widely used for phase mapping and high throughput screening of combinatorial films. The X-ray microbeam is generated by first applying a graded multilayer mirror or monocapillary optics for collecting the divergent X-ray beam generated from the X-ray tube and then aperturing it down to a diameter as small as 10 um. For data collection, this system uses a photon-counting multiwire area detector without intrinsic background. A prototype instrument specially designed for combinatorial libraries characterization was jointly developed at ARACOR and Intermix recently. This work was carried out with a laboratory X-ray source to implement the combined X-ray diffraction and fluorescence techniques. But there are some disadvantages with the X-ray microbeam technique. For example, often one needs accurate lattice parameter determination of < 5 μm spatial resolution, so the use of a synchrotron X-ray beamline is beneficial.

The synchroton technique offers unparalleled sensitivity and spatial resolution. An integrated Hard X-ray Nanoprobe (HXN) was jointly developed by a team from Argonne National Laboratory (ANL) and Xradia Inc. and reported by Eric Isaacs from ANL (R&D 100 Award). The HXN is the first system to integrate X-ray fluorescence, X-ray diffraction, and full-field imaging exchangeable into a single instrument. This integration allows fast acquisition of full-field tomographic images combined with X-ray fluorescence and/or X-ray diffraction characterization in an *in situ* manner. The HXN offers the combination of superior hard x-ray spatial resolution with high elemental and strain sensitivity and operates at atmospheric pressure or in a vacuum.

Techniques used to visualize and to identify X-ray spectral data of combinatorial libraries are important for mapping phase diagrams. Takeuchi’s research group at University of Maryland has been actively developing techniques for managing and visual inspection of x-ray spectra of combinatorial films [54–56]. For example, the *MATLAB* software has been used to efficiently organize the data and create various plots from which one can obtain important information regarding structural and phase changes across the combinatorial libraries. The *Spectrum Scroller* software allows users to rapidly view hundreds of XRD spectra and corresponding compositions in a matter of minutes. The *Angle Scroller*, allows users to inspect the intensity data for all of the spectra simultaneously, but only one angle at a time. Long and Takeuchi [55] have developed a cluster analysis procedure for identifying structural phases and their distribution in thin film composition spreads. In principle, using this method, the tedious analysis and classification of hundreds of spectra is reduced to the analysis of a small number of spectra. Long et al. [56] also applied the technique called non-negative matrix factorization (NMF) to analyze the phases present by deconvolving a large number of non-negative spectral patterns into a smaller number of non-negative basis patterns. Currently improvement of these techniques continues.

#### 2.2.5 Literature Examples of Combinatorial Thin Film Phase Diagrams

**Fig. 6:**
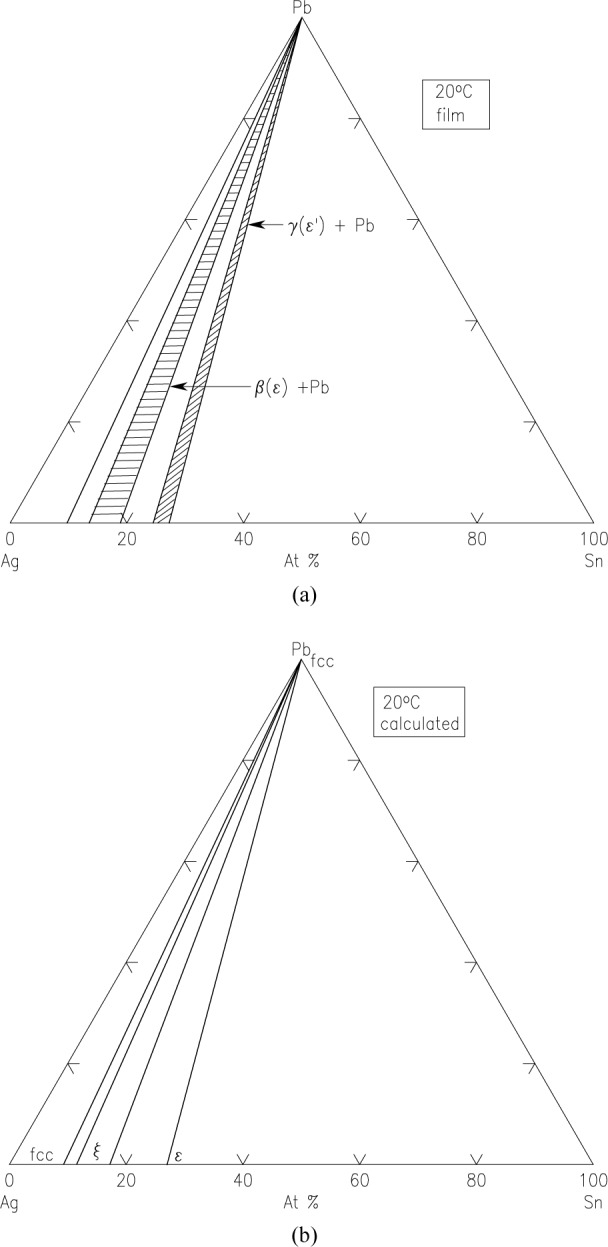
Phase diagrams of the Ag-Pb-Sn system at 20 °C, (a) obtained by the combinatorial thin film approach, (b) calculated diagrams [5,6].

**Fig. 7:**
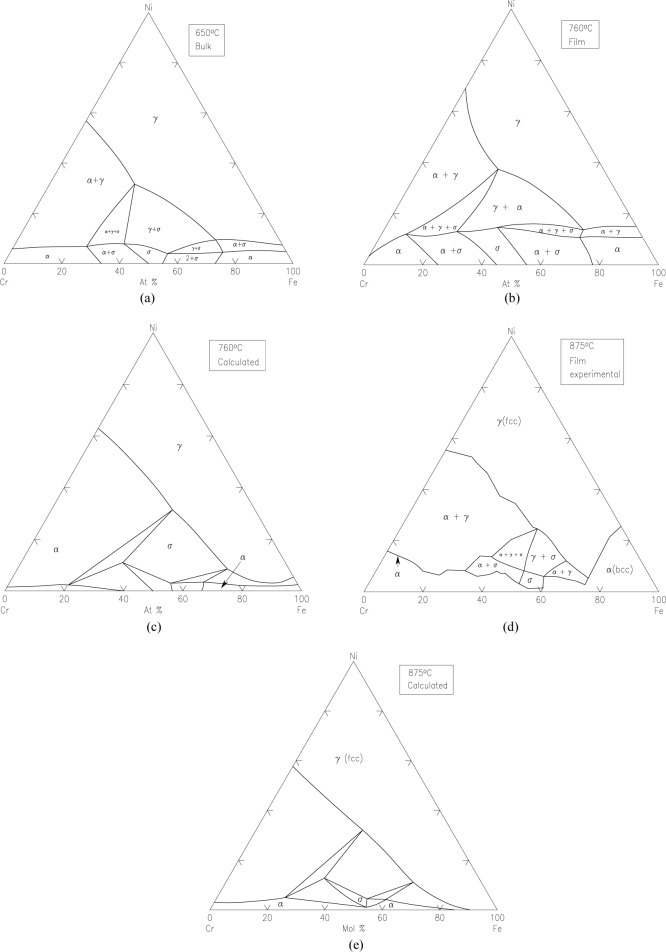
Phase diagrams for the Fe-Cr-Ni system obtained by the combinatorial thin film approach: (a) bulk diagram at 650 °C; (b) film diagram at 760 °C [8]; (c) calculated diagram at 760 °C [5]; (d) experimental film diagram at 875 °C [60,61]; and (e) calculated diagram at 875 °C.

**Fig. 8:**
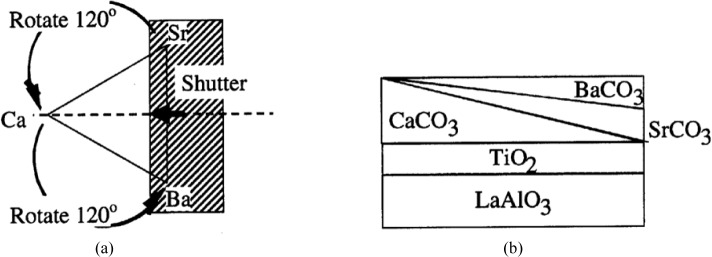
The PLD shutter system and the resultant precursor or layer profiles (viewed edge-on) for preparing the (Ba_1−x−y_Sr_x_Ca_y_)TiO_3_ (0<x<1 and 0<y<1) ternary composition spread film [62].

**Fig. 9:**
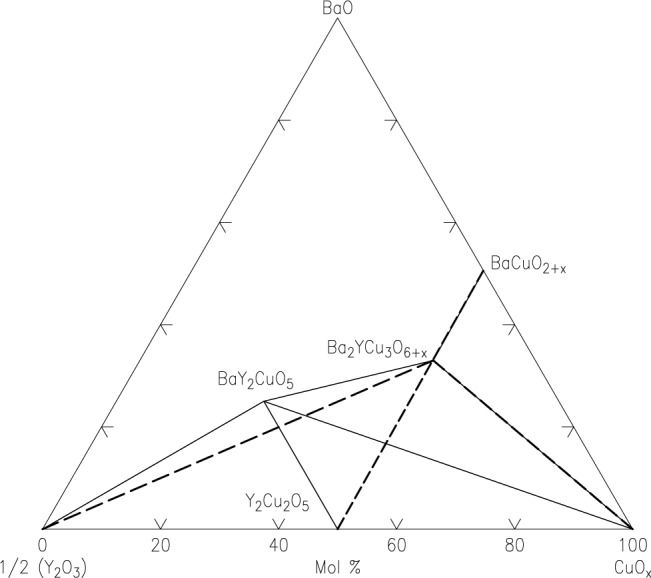
Phase relationships constructed based on results from the combinatorial synthesis approach (BaCuO_2_, Y_2_O_3_ and CuO as targets on substrates SrTiO_3_ and RABiTS), HTXRD experiments on a film with nominal composition of Ba:Y:Cu ≈ 1:2;1, and X-ray diffraction studies of the BaY_2_CuO_5_ bulk sample; dotted-lines (T < 800 °C, ≈ 100 *Pa p_O2_*) and solid lines (T ≈ 800 °C to 850 °C (≈ 100 *Pa p_O2_*)) [63].

Although the earliest reported method of preparing combinatorial thin films can be traced back to 1965 (Kennedy et. al. [8]), to date, only a small number of thin film phase diagrams have been reported. The following summarizes the systems investigated:

1. Ag-Pb-Sn System
  Boettcher et al. [6] tested Ag-Sn and Au-Sn binary systems first using the thin film approach, and then mapped the phase diagram of the ternary system Ag-Pb-Sn system at 20 °C. Although the isothermal section at 20 °C is not experimentally measured for comparison, the phase diagram was calculated from modeling [5]. The experimental and the calculated diagrams agree reasonable well with each other, as seen in Fig. 6a and Fig. 6b, respectively.
2. Fe-Cr-Ni System
  This system is the basis of a wide variety of commercially important alloys in stainless steel. The Fe-Cr-Ni system was first studied by Kennedy et al. [8]. They evaporated pure Cr, Fe, and Ni from three ingots using EB-PVD to create a thin film with varying ternary compositions on a substrate. The substrate was heated to 760 °C and all phases formed were equilibrium ones. The compositions were determined using X-ray fluorescence and the crystal structures were determined using X-ray diffraction. The experimental isothermal sections of the Fe-Cr-Ni system have been determined at 650 °C (Fig. 7a). Although an experimental isothermal section at exactly the same temperature as the bulk equilibrated alloys was not reported, a calculated diagram [5] shows that the overall topology of the phase diagram and the number of equilibrium phases are consistent, despite some differences in the locations of the phase boundaries. These differences may be attributed to errors in XRF determination. The diagrams of the bulk system at 650 °C (Fig. 7a), film system at 760 °C (Fig. 7b) and the calculated diagram at 760 °C (Fig. 7c) are qualitatively comparable, but quantitatively somewhat different because of the different isothermal temperatures.
  Later the phase diagrams of the Fe-Cr-Ni system were also studied by Rar and Specht [57–61] at 825 °C and at 875 °C. The alloy libraries were prepared by depositing three layers of Cr, Fe, and Ni metals with a linear thickness gradient rotated by 120° for each layer. These layers were interdiffused and alloyed by annealing in vacuum. They described a technique based on XRD and fluorescence using synchrotron radiation to analyze efficiently a large number of material compositions, to characterize the structure and composition of isothermal sections of the phase diagram, and to construct contour maps of lattice parameters. Approximately 2500 compositions were examined in a single experiment taking about 4 h. From the integrated patterns, phases were identified for the construction of phase diagrams. They also analyzed texture of the films based on intensity distribution; the diffraction pattern identifies both epitaxial and randomly oriented phases [59]. With this technique, they were able to determine equilibrium phases as well as metastable ones. Their phase diagram prepared from annealing experiments at 875 °C was compared to a calculated one at the same temperature (Fig. 7d and Fig. 7e) (an experimental diagram using bulk materials was not available). There appear to be differences at 875 °C, particularly the shift of the phase boundaries.
3. (Ba_1−x−y_Sr_x_Ca_y_)TiO_3_ (0<x<1 and 0<y<1) ternary composition spread film
  Xiang et al. [62] prepared a continuous-phase diagram by fabricating an epitaxial thin-film ternary composition spread of (Ba_1−x−y_Sr_x_Ca_y_)TiO_3_ (0<x<1 and 0<y<1). The precursor film was deposited in a high vacuum PLD system (10^−7^ torr). The system used an *in situ* shutter that was computer-controlled to move across the surface of the substrate (to give a linear thickness gradient). The substrate used was LaAlO_3_ cut into the shape of a triangle with the height of 25.4 mm; a layer of TiO_2_ was first deposited uniformly on the substrate. By moving the shutter combined with a rotation of 120°, a linear thickness gradient of CaCO_3_ (0 Å to1225 Å), BaCO_3_ (0 Å to 1647 Å) and SrCO_3_ (0 Å to 1475 Å) was produced. Figures 8a and 8b give the PLD shutter system and the resultant precursor or layer profiles, viewed edge-on. After high temperature annealing, which removed the CO_2_, a high quality film of the (Ba_1−x−y_Sr_x_Ca_y_)TiO_3_ system was obtained. X-ray diffraction patterns indicated single phase properties of the solid solution region. Therefore the combinatorial approach appears to be an excellent approach for investigating solid solution regions of this type.
4. Ba-Y-Cu-O diagram [63,64]
  Application of the second-phase precipitation method for flux pinning in coated conductors requires accurate knowledge of the nature of phases that are compatible with Ba_2_YCu_3_O_6+x_ under relevant processing conditions. Figure 9 summarizes phase relationships for the Ba-Y-Cu-O system as deduced from our combinatorial experiments on compositional spreads [63], *in situ* XRD experiments on BaF_2_-based films, X-ray diffraction studies of a BaY_2_CuO_5_ bulk sample, and our previous phase equilibrium work [64]. In this diagram, dotted tie-lines refer to T < 800 °C (*p_O2_* ≈ 100 *Pa*) whereas solid lines corresponds to the temperature range between 800 °C to 850 °C (*p_O2_* ≈ 100 *Pa*). Clearly, the processing temperature appears to be the most critical parameter that controls formation of the Ba-Y-Cu-O phases exhibiting flux-pinning properties in both thin films and bulk materials. Therefore, for the BaF_2_ process, the research suggests that temperatures above 800 °C (*p_O2_* ≈ 100 *Pa*) are necessary for the formation of BaY_2_CuO_5_ flux pinning centers.

#### 2.2.6 Other Thin-Film Phase Diagramming-Related Studies

Many authors have attempted to use approximate phase diagrams as a basis for exploring a variety of properties. These are essentially composition diagrams or processing diagrams portrayed using a phase diagram framework, and are sometimes referred to as a “semi phase diagram” or a “synthesis diagram.” Such diagrams summarize available information and may include both equilibrium and non-equilibrium data. In this sense, although these differ from the equilibrium phase diagram, and may not be thermodynamically self-consistent, such diagrams may have important practical aspects related to a given set of conditions.

Lewis et al. [65] applied combinatorial methods to determine hydrogen sorptive properties of phases in the system LiNH_2_-MgH_2_-LiBH_4_. Reaction phase diagrams of pseudo four-component oxides Li-Ni-Co-Ti-O have been determined by Fujimoto et al. [66] to explore the single-phase composition of the layer-type compound (LiNi_0.8−x_Co_0.2_Ti_x_O_2_ (0<x<0.1)) and the evaluation of charge-discharge properties by the combinatorial electrode evaluation system. Downey and van Dover used a composition spread approach to explore novel thin film amorphous oxides and to develop novel materials for optical amplifiers [67]. Tsui et al. used a phase diagram as a basis to explore epitaxial processes in doped magnetic semiconductors [68]. Koinuma et al. used combinatorial CeO_2_-Al_2_O_3_-HfO_2_ films for screening high dielectric constant (high-k) materials as prospective gate compounds for MOSFET transistors [69]. Others have studied transition alloy superconductors [70] and determined the magnetic-optical Kerr effect in the thin film single solid solution phase region of (Ba,Sr,Ca)TiO_3_ [62]. Chang et al. [71] fabricated Ni-Ti-Pt ternary metal gate thin film libraries on HfO_2_ using magnetron co-sputtering to investigate flatband voltage shift, work function and leakage current density variations.

#### 2.2.7 General Factors Influencing the Construction of Film-Based Combinatorial Phase Diagrams

Important issues relevant to the construction of reliable diagrams based on experiments with thin film materials can be summarized by the following:

1. *Interactions/reactions between substrate and film.* The substrate on which the film is deposited can have a very strong effect on the phase formation in the combinatorial film, which complicates the phase diagrams. The composition could be modified, resulting in a multicomponent system different from the one that was intended. Therefore one of the principal criteria for the selection of a substrate material is to minimize alloying between the substrate and the deposited film at the temperature of deposition, or at any subsequent annealing temperature.
2. *Adherence between the deposited film and substrate.* There should be good adherence between the deposit film and substrate, particularly for brittle deposits. Substrate materials include Si, stainless steel, and Mo foil. For good adherence, surface roughening, such as produced by carefully controlled sandblasting, might be a possible choice for promoting adherence.
3. *Reaction of film with environment.* Possible reactions of the film with the environment (oxygen, carbon dioxide, hydrogen) need to be avoided. These reactions may result in unwanted phases as well as adding kinetic barriers to the formation of equilibrium phases.
4. *Lattice mismatch with substrate and coherency strain effects* [72,73]. Phase diagrams can be significantly different under conditions of positive, zero or negative strain. These types of effect were documented by Li et al. [72] in the study of the PbTiO_3_-PbZrO_3_ system. Phase field modeling can be used to define the overall topology of phase diagrams.
5. *Effects of temperature.* Temperature has a great effect on phase formation. It is important to calibrate the temperature of the deposition system at the surface of the film where the deposition takes place. For example, in a PLD system, the thermocouple in most cases is situated under the substrate, so one needs to estimate the actual temperature of the film, based on the thermocouple temperature. In addition, because the PLD process produces highly energetic material, the apparent temperature of phase formation may be lower than the temperature reported for bulk phases. As a result of such kinetic effects, thin-film deposition processes may lead to the presence of phases which would be regarded as metastable relative to bulk phases at a given temperature.
6. *Effect of oxygen partial pressure.* It is necessary to control oxygen partial pressure (or partial pressure of other relevant volatiles) well throughout experiments. For oxides, an appropriate oxygen background pressure must be maintained during PLD deposition. For metal or semiconductor, alloys, oxygen contaminants must be avoided.
7. *Effects of texture.* Severe texturing may affect the interpretation of x-ray patterns by changing the appearance of x-ray patterns, which in turn may lead to misidentification of phases or to an insufficient number of peaks for phase identification. Texture can also change properties of materials, energy of reaction and energy of phase formation.
8. *Surface energy effects.* Because the film particles are essentially of nano size, there is substantial surface area leading to surface energy effects which may result in apparent tie-line relationships that are different from bulk phase diagrams.
9. *Phase boundaries.* Phase/structure identification needs to be performed well for localized compositions in order to construct the one, two- or three-phase regions, otherwise phase boundaries would be poorly defined.
10. *Equilibrium vs. non-equilibrium.* Non-equilibrium states often exist on films. As a result, the diagrams that one obtains may not be the same as that of the equilibrium diagrams. One needs to pay attention to whether equilibrium phases are formed in the thin films, particularly if the thin-film substrates are not heated to the temperature of interest during deposition. Sometimes several annealing trials may be needed to produce the equilibrium phases.

#### 2.2.8 Specific Measurement Considerations for Determining Reliable Combinatorial Film Diagrams

There are several limitations for combinatorial film techniques at the present state-of-the-art. For the CCS technique with PLD, one often needs to use a 3 inch diameter substrate. Large substrates other than Si wafers are sometimes too expensive or not available for many applications that require specific types of substrates. Another limitation of the ternary co-deposition technique is that the film cannot include pure elements (as end-members of a binary or a ternary system) or binary elements (of a ternary system) because with the PLD technique it is not practical to produce compositions near the end members. In other words, certain ranges of the film cannot be covered, which sometimes are critical for mapping tie-line relationships. Despite these limitations in preparing combinatorial film libraries, if one takes careful measures, one should be able to obtain reliable diagrams over most of the desired compositional space. Specific measurement and instrumental issues that are related to the general factors described above, are summarized as follows:

1. *Compositions of films.* One needs reliable tools to determine accurate compositions of the films. Often compositions are not linearly proportional across the film, so one needs the combined information of x-ray and compositional analysis to construct a final reliable diagram.
2. *Temperature calibration of the substrate.* Depending on the methods of synthesis (PLD, sputtering, etc.), accurate effective temperature and energy of the deposition medium should be determined.
3. *Temperature profile across film.* The temperature profile across the film (produced by chamber substrate heaters) must be homogeneous. If the film is formed under non-isothermal conditions, some regions on the film will have higher temperature which may cause melting to occur. If incongruent melting occurs, the phases crystallized are likely to be different from the corresponding subsolidus phase, and liquid migration may change the local composition. It is also important to avoid local melting due to the possible production of an amorphous state. These factors would complicate the determination of homogeneity regions and tie-lines and could lead to incorrect diagrams. As a summary, different phases may occur at different temperatures, therefore if the temperature is not homogeneous throughout the film, the diagram obtained would be a composite diagram.
4. *Substrate compatibility.* Unless the substrate is chemically compatible, there could be more components than intended. The choice of substrate depends on the targets used. For example, for finding solid solution regions of perovskites, the substrate should have similar structure to that of a perovskite (if possible, with similar cell parameters). This could also promote better property screening.
5. *Unwanted gases.* Vapor-deposited films are often badly contaminated with unwanted gases. One should minimize the presence of these gases by using high evaporation rates and by maintaining high vacuum (for example, < 6.67×10^−6^ Pa).
6. *Diffraction techniques.* One needs to optimally apply effective x-ray techniques with good resolution. Software used to identify phases of the patterns depends on resolution. It would be beneficial to have synchrotron radiation for high resolution for separating overlapping peaks from films and substrate; particularly a system that can perform simultaneous diffraction and fluorescence experiments would be ideal. One can develop portable synchrotron sources in the laboratories.
7. *Lattice parameter determination.* It would be advantageous, again, to use the synchrotron beamline if possible for obtaining accurate lattice parameter measurement at < µm spatial resolution.
8. *Melting point measurements.* Accurate melting point measurements corresponding to different locations (compositions) in the combinatorial spread films are important so that it can be established in advance that no part of the film undergoes melting at the temperature of the film deposition process. A few compositions should be prepared to outline melting behavior prior to the actual combinatorial deposition.
9. *Cooling rate of film.* If the substrate is cooled slowly in the chamber, phase transitions may occur during cooling. If the film is quenched instead, then after film removal, subsequent annealing may be important to ensure that equilibrium structures form. The optimal procedure will have to be determined for each system as part of the investigation.
10. *Software and strategy for phase analysis.* It is deemed important to have reliable software and strategy for analysis of diffraction patterns. There will be an enormous amount of diffraction data being generated per system. The software needs to be able to perform the phase identification process as fully automatically as possible (with pointers to various single crystal and powder diffraction crystallographic databases). At the same time we also need software that is capable of organizing and storing the diffraction data of the combinatorial film in a well-designed format for future retrieval and reference use.
11. *Crystallographic databases.* It is important to use fully evaluated and informative databases for phase analysis. Examples of these databases at the Power Diffraction file (PDF) [74], Inorganic Crystal Structure Database (ICSD) [75], and the Cambridge Structural Database (CSD) [76]).

## 3. Conclusions

Diffusion couples and multiples constitute a combinatorial synthesis tool for the parallel generation of bulk intermetallic compounds and solid solutions. The key advantage of diffusion couples/multiples is the formation, in principal, of all equilibrium intermetallic compounds and solid solution phases, with a complete range of all single-phase compositions for binary and ternary systems. The diffusion-multiple approach can increase the efficiency of phase diagram mapping by orders of magnitude, facilitating the availability of phase diagrams for alloy design. For the development of existing alloy systems, such as superalloys, the diffusion-multiple approach can be applied to obtain critical data to improve the reliability of the thermodynamic database. One particular area which has room for improvement is the spatial resolution of the micro-analytical techniques used. Errors attributable to extrapolated interface compositions can then be minimized. Also, although the diffusion multiple technique is a unique and powerful tool in materials science studies, it is most useful in conjunction with several analytical methods for enhancing the reliability of the results.

The film combinatorial technique is an efficient, state-of-the-art technique for studying phase relationships of material systems in thin film form. The diagrams being constructed depend on various experimental conditions (and to some extent, the analytical tools). A complete ternary alloy deposition can easily be made in a day, however, the determination of composition, structure and tie-line relations is still time-consuming and we need rapid and efficient methods for determining them. Precise stoichiometric control over a small region is needed to identify important phase boundaries and narrow-phase regions. Considerations for determining reliable combinatorial film diagrams include the interaction of film with substrate, adherence between the deposited film and substrate, reaction of film with environment, lattice mismatch to substrate and coherency strain effects, temperature effects, effects of oxygen partial pressure, texturing effect, and surface energy effects. Improvements central to the effort for obtaining reliable phase diagrams include homogeneous temperature profile across film, better composition determination and better software routines for identifying phases. The availability of standard reference patterns in crystallographic data bases (such as the PDF, the ICSD, and the MSD) for characterizing phases present in the system as well as for potential interaction products of film with substrate in the ICDD and ICSD data bases is extremely important for the interpretation of phase assemblages.

An important trend in phase diagram determination is the increasing interplay of modeling and experiments. By combining experimental film diagram data with calculation results, one can obtain more reliable diagrams. Therefore comparison of experimental diffusion couples/multiples and thin-film combinatorial diagrams with those obtained via a modeling approach should become a standard practice.
